# Anti-HIV-1 ADCC and HIV-1 Env Can Be Partners in Reducing Latent HIV Reservoir

**DOI:** 10.3389/fimmu.2021.663919

**Published:** 2021-04-30

**Authors:** Poonam Suryawanshi, Rajani Bagul, Ashwini Shete, Madhuri Thakar

**Affiliations:** ^1^ Deaprtment of Immunology and Serology, ICMR-National AIDS Research Institute, Pune, India; ^2^ Faculty of Health Sciences, Symbiosis International University (SIU), Pune, India

**Keywords:** anti-HIV antibodies, latent HIV, ADCC, HIV Env, HIV

## Abstract

**Background:**

Persistence of HIV reservoir even in suppressive ART is the key obstacle in HIV-1 cure. We evaluated the ability of HIV-1 C Env to reactivate the latently infected resting memory CD4 cells and the ability of polyclonal HIV antibodies mediating ADCC to lyse the reactivated targets.

**Methodology:**

HIV-1 antibodies from 25 HIV infected individuals (14 ADCC responders and 11 non-responders) were tested against the Env-C reactivated primary cells; CD4+ and CD4+CD45RO+ memory T cells in the presence of autologous or heterologous effector cells using multicolor flow cytometry. The frequencies of p24+ve target cells were measured to determine the reactivation and antibody mediated lysis.

**Results:**

Increase in the frequency of p24 expressing cells (P < 0.01 in all cases) after Env-C stimulation of target cells indicated reactivation. When these reactivated targets were mixed with effector cells and HIV-1 antibodies, the frequencies of p24 expressing targets were decreased significantly when the ADCC mediating antibodies (P < 0.01 in all cases) were added but not when the antibodies from ADCC non-responders or HIV negative individuals were added. In parallel, the NK cell activation was also increased only when ADCC mediating antibodies were added.

**Conclusion:**

The study showed that the HIV-1 Env could act as latency reversal agent (LRA), and only ADCC mediating antibodies could lyse the reactivated HIV reservoirs. The short stimulation cycle used in this study could be useful in testing LRAs as well as immune mediated lysis of reactivated reservoirs. The observations have further implication in designing antibody mediated immunotherapy for eradication of latent HIV reservoir.

## Introduction

Despite viral suppression in HIV-1 infected individuals on antiretroviral treatment (ART), the virus persists in long-lived latent reservoirs (1), the elimination or reduction of which is a major hurdle in achieving HIV cure (2). A classical ‘shock and kill’ approach is being used to reduce HIV reservoirs. The shock is to reactivate the latently infected cells, and the kill involves killing of these reactivated cells expressing HIV antigens by immune-mediated mechanisms. In addition to the HIV-1 cytotoxic T cells and anti-HIV broadly neutralizing antibodies (bNAbs) (3, 4), the anti-HIV antibodies mediating Antibody Dependent Cell Cytotoxicity (ADCC) are also thought to play a role in reducing reactivated latent reservoir as these antibodies induce killing of antigen expressing infected cells by bridging between the antigen presenting cell and innate effector cells such as natural killer (NK) cells or monocytes (5–7). The presence of anti-HIV ADCC mediating antibodies was also shown to be associated with slow HIV disease progression (8) and has been implicated as an immune correlate in the moderately successful HIV-1 RV144 vaccine trial (9).

The numbers of latency reversal agents (LRAs) have shown sufficient reactivation of the latent reservoir, and a few are in clinical trial (10). However, LRAs alone were insufficient to boost the immune response against HIV, underscoring the need of immunological interventions for efficient elimination of the reactivated latent reservoir (11). We have previously shown that the HIV-1 Env can reactivate the HIV positive primary CD4+ T cells and also improve HIV specific CTL response (12). However, the ability of ADCC mediating anti-HIV antibodies to lyze the Env reactivated reservoirs has not been studied. Also the ability of HIV antigens such as Env for latency reversal has not been assessed. Hence in the present study, we assessed the ability of anti-HIV ADCC antibodies to induce NK cell mediated lysis of Env stimulated HIV reservoirs. We used the primary cell model with a short stimulation cycle for reactivation of the infected cells using intracellular p24 expression as a marker of reactivation. The reduction in the p24 expressing cells in the presence of ADCC mediating antibodies and effector cells was used as an indicator of lysis of the reactivated cells. We showed that the Env antigen can reactivate the HIV infected memory CD4 cells which were efficiently lysed by the ADCC mediated lysis. The short stimulation cycle model used in this study will be useful in latency reversal assessment of various LRAs also.

## Materials and Methods

### Study Participants

Twenty-five (age: 30–48 years, Male: five, Female: 20) ART naive asymptomatic HIV-infected individuals were identified from our previous study (13) as ADCC responders (N = 14, showed HIV Env C specific ADCC response) and ADCC non-responders (N = 11) and enrolled in the present study. The CD4 counts of these participants ranged from 410 to 1,316 cells/mm^3^ (median: 576 cells/mm^3^) at the time of the enrollment in the present study. Additionally, 11 HIV uninfected healthy controls (age: 21–46 years, Male: five, Female: six) were also enrolled to determine non-specific antibody reactivity. Furthermore, five ART naïve HIV infected individuals were enrolled in the study for assessing reduction of latently infected cells using resting memory CD4 cells (CD4 count—median: 625, range: 523–825; Age—Median: 42 years range: 29–56 years; Male: two, Female: three). Thirty ml whole blood was collected from each individual in heparinized vaccutainers. The peripheral blood mononuclear cells (PBMCs) and plasma were separated by density gradient centrifugation according to standard protocols and stored till further use. The study was approved by the Institutional ethical review board (NARI/EC/2015-13), and the study participants provided written informed consent at enrollment.

### Antibody Purification

The IgG antibodies were eluted from the plasma samples of the enrolled participants using NAb Protein G Spin Kit (Thermo Fisher Scientific Waltham, MA) according to the manufacturer’s instructions and desalted using the Spin Desalting Columns. The concentration of the purified antibody was determined by Nanodrop (ND1000 V3 7.1, Thermo Fisher Scientific), and the E antibodies were stored at −20°C until use. The purified IgGs isolated from the ADCC responders, ADCC non-responder participants, and HIV uninfected controls are referred as ADCC IgGs, non-ADCC IgGs, and HIV negative IgGs respectively in the manuscript.

### Flow Cytometry-Based Latently Infected Cell Reduction Assay

#### Using ACH2 Cells as a Model of Latently Infected Cells

**Figure 1:**
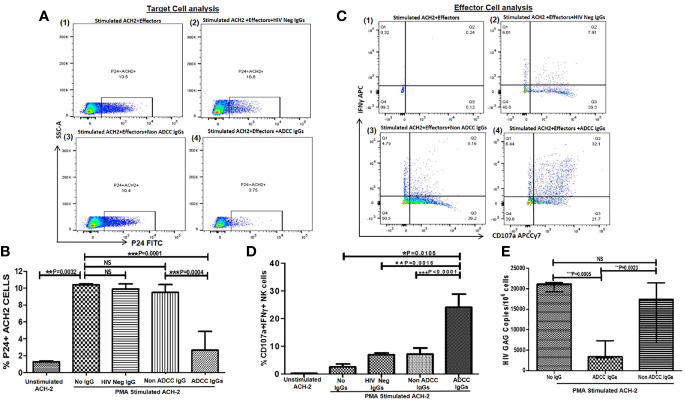
ADCC activity against reactivated ACH-2 cells. **(A)** The representative dot plots show % p24+ACH-2+ cells (X axis) in (1) PMA stimulated ACH2 with PBMCs (as a source of effector: Nk cells) without antibody (2), after addition of HIV Neg IgGs or (3) non-ADCC IgGs or (4) ADCC IgGs. **(B)** The bar diagram shows frequency of the p24+ACH2 cells (Y axis) in unstimulated ACH-2 control, after PMA stimulation, after addition of HIV Neg IgGs (n = 5), non-ADCC IgGs (n = 11), and ADCC IgGs (n = 14)(X axis). **(C)** The representative dot plots show NK cells (effectors from PBMC of HIV negative healthy individual) expressing IFN*γ* (on Y axis) and CD107a expression (on X axis) in (1) PMA stimulated ACH2 with PBMCs without antibodies (2), after addition of HIV Neg IgGs or (3) non-ADCC IgGs or (4) ADCC IgGs. **(D)** The bar graph shows frequencies of CD107a and INF*γ* secreting NK cells (on Y axis) in unstimulated ACH-2, after PMA stimulation, after addition of HIV Neg IgGs, non-ADCC IgGs or ADCC IgGs (on X axis). **(E)** The bar diagram shows HIV gag DNA copies per million cells in ACH-2 cells (on Y axis) after PMA stimulation, after addition of ADCC IgGs and non-ADCC IgGs (on X axis). NS, not significant.

First we assessed the ability of the purified ADCC antibodies to lyse reactivated ACH-2 cells, the cell line mimicking latent HIV reservoir. The reduction in the frequency of p24 positive cells after addition of antibodies was used as a marker for lysis of reactivated ACH-2 cells (14, 15) Briefly, the ACH-2 cells were stimulated with 10 nM of Phorbol-12-myristate-13-acetate (PMA, Sigma) for 24 h. Then ACH2 cells were washed with 10% FBS + RPMI medium to remove free virions secreted in the medium, if any. These PMA stimulated ACH-2 cells and the PBMCs isolated from an HIV negative healthy individual (as a source of NK cells) were incubated at target: Effector ratio of 1:10 with purified ADCC IgGs (n = 14), non-ADCC IgGs (n = 11), and HIV negative IgGs (n = 5) for 5 h at 37°C in 5% CO2 with anti CD107a APC antibody (BD Biosciences, San Jose, CA), BFA (5 µg/ml; Sigma), and Monensin (6 µg/ml; Sigma). After incubation, the cells were stained with anti-CD3 PETR, anti-CD8 PECy5, anti-CD4 PE (BD Biosciences, San Jose, CA), and anti-CD56 PECy7 (BioLegend San Diego, CA), for 30 min at room temperature. The cells were fixed, permeabilized, and stained with anti P24 FITC (KC-57 FITC; Beckman Coulter Brea, CA) and anti IFN-γ APC (BD Biosciences, San Jose, CA) for 30 min at room temperature. Cells were again washed, fixed with 1% Formaldehyde and acquired within 24 h to get 100,000 gated lymphocyte events on FACSAria I (BD Biosciences). The data was analyzed using FACS Diva software V4.0 (BD Biosciences). The gating strategy for ACH-2 and NK cells is shown in **Supplementary Figure 1**. The activation of NK cells was also assessed for expression of degranulation marker CD107a and intracellular secretion of IFNγ. All the experiments were run in triplicate, and the results are expressed as median of all experiments. The representative dot plots for %p24+ACH-2+ cells in PMA stimulated ACH2 and NK cell activation have been shown in **Figures 1A, C** respectively.

#### Quantification of HIV Provirus DNA

Reduction in HIV proviral DNA after incubation with ADCC antibodies is also an indication of lysis of the reactivated ACH2 cells. To test this, the DNA was extracted from the cell mixture of ACH2 + effectors with or without ADCC/non-ADCC IgGs using a commercial kit (Qiagen, Hilden, Germany). Total HIV DNA was quantified by qPCR using a primer set targeting the HIV *gag* gene (HIV GAG forward primer 5^0^-ACCCATGTTTACAGCATATCAGAAG-3^0^, HIV GAG reverse primer 5^0^-GCTTGATGTCCCCCTACTGTATTT-3^0^) and housekeeping gene *β* Actin (*β* Actin forward 5^0-^CACCAACTGGGACGACAT-3^0^, *β* Actin reverse 5^0^-ACAGCCTGGATAGCAACG-3^0^). All samples were assayed in duplicate, and qPCR assays were performed on an ABI 7900HT instrument. Cycling conditions were as follows: 50°C for 2 min followed by 95°C for 10 min for polymerase activation, followed by 40 cycles of 95°C for 15 s and 60°C for 1 min. To generate a standard curve a latently HIV infected T cell line ACH-2 containing one copy of integrated HIV DNA per cell was used (NIH Reference Reagent Program). HIV Gag and *β* Actin levels were quantified using respective primers, and standard curve was plotted.

#### Using HIV ENV C-Activated HIV Infected CD4+ T Cells

**Figure 2:**
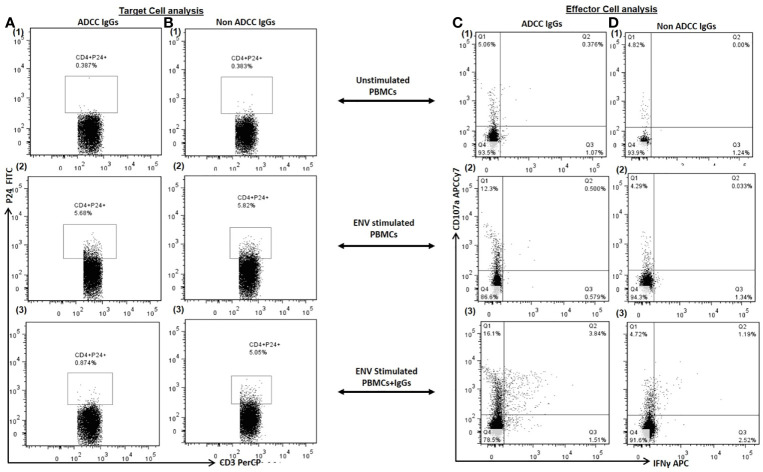
Target Cell Analysis. The representative dot plots show frequency of p24+CD4+ cells (P24 on Y axis and CD3 on X axis) in (1) unstimulated PBMCs (as a source of both target: primary CD4+ T cells and effector: Nk cells) (2), Env stimulated PBMCs and (3) after addition of autologous ADCC IgGs in Env simulated PBMCs (Column **A**) and after addition of autologous non ADCC IgGs (Column **B**). Effector Cell Analysis. The representative dot plots show CD107a (on Y axis) and IFN*γ* (on X axis) secreting NK cells in (1) unstimulated PBMCs (2), Env stimulated PBMCs and (3) after addition of autologous ADCC IgGs in Env simulated PBMCs (Column **C**) and after addition of autologous non ADCC IgGs (Column **D**).

Next, we assessed the lysis of Env-stimulated HIV infected CD4+ cells by ADCC. For this, PBMCs (as source of HIV infected CD4+ cells and NK cells) from ADCC responders (n = 14) and non-responders (n = 10) were stimulated with HIV-1 Env-C peptide pool (cat no.9499, NIH AIDS Reagent Program) at a final concentration 5 µg/ml or left unstimulated for 3 h at 37°C in 5% CO_2_. The Env was removed by washing the cells with 10% FBS + RPMI medium to ensure minimal chances of bystander killing of target cells. The cells were then incubated with autologous purified ADCC IgGs and non-ADCC IgGs for 5 h, and the frequencies of p24 positive CD4+ T cells and activated NK cells were measured using multicolor flow cytometry as described earlier in the manuscript. The gating strategy for the target and the effector cells is detailed in **Supplementary Figure 2**. The representative dot plots of the autologous CD4+p24+ cells and NK activation before and after the addition of antibodies have been shown in **Figure 2**.

#### Using HIV ENV C-Stimulated HIV Infected Resting Memory CD4+ T Cells (Latent Reservoir)

Further we assessed the reduction in Env C-reactivated the latent (resting memory CD4+ cells) by ADCC using the PBMCs from five ART Naïve HIV-1 infected individuals. The CD4+CD45RO+ cells (resting memory CD4+ cells) were sorted as a source of latent reservoir, and NK cells were sorted as a source of effector cells. For this the PBMCs were stained for anti-CD4 APC, anti-CD8 BUV737, anti-CD45RO PECy5.5 (all from BD Biosciences, San Jose, CA) for 30 min at room temperature. The lymphocytes were gated on the basis of their forward and side scatter. From theCD4+CD8− cells, the CD45RO+CD4+ cells were selected as resting memory CD4 cells and collected in one tube. The CD4− and CD8− cells were collected into the second tube as a source of effector cells. The purity of sorted cells was >90% in all cases.

The sorted resting memory CD4 cells were reactivated with HIV-1 Env C peptide pool at a final concentration 5 µg/ml for 3 h at 37°C and 5% CO_2_. After washing, the reactivated resting memory CD4 cells and CD8 depleted autologous effector cells were cultured at 1:1 ratio in the presence of purified selected ADCC IgG/non-ADCC IgGs/HIV negative IgGs at a concentration 1 mg/ml for 5 h at 37°C along with anti CD107a APCCy7 antibody (BD Biosciences, San Jose, CA), BFA (5 µg/ml; Sigma), and Monensin (6 µg/ml; Sigma). After 5 h incubation, cells were stained as described earlier in the manuscript. The gating strategy for resting memory CD4+cells and NK cells is shown in **Supplementary Figure 3**. The Representative sample dot plots showing frequency of P24+CD45RO+CD4+cells and NK cell activation have been shown in **Figures 4A**, **E** respectively.

### Statistical Data Analysis

The data was analyzed using statistical software Graph Pad prism V5.0 (GraphPad Software, San Diego, CA). The non-parametric Wilcox’s matched paired t test was used to evaluate difference between different parameters in paired samples. Differences in variables between the study groups were evaluated using Mann–Whitney U test, and Spearman correlation was used for correlation analyses. Data are reported as median with Interquartile range. The P value <0.05 was considered as significant.

## Results

### ADCC Mediating Antibodies Lyse the Reactivated ACH-2 Cells

The median frequency of p24+ ACH-2 cells was significantly increased from 1.44 to 10.5% (range: 10.4–10.8%) after 24 h of PMA stimulation indicating reactivation of the ACH 2 cells. This frequency decreased significantly after addition of ADCC IgGs (median: 2.65, range: 0.11 t–7.26%) (P = 0.0001) but remained unchanged after addition of HIV Neg IgGs (median: 9.9, range: 9.6–10.7%) and non-ADCC IgGs (median: 9.5, range: 5.5–10.8%) which was significantly higher as compared to frequencies after addition of ADCC IgGs (P = 0.0004) (**Figure 1B**).

In parallel, the ADCC IgGs induced considerably higher activation of NK cells (median: 24.1, range: 13.4–34.1%) as compared to without IgG control (P = 0.0105)(median: 2.5, range: 2.1–3.6%) or the NK cell activation after addition of HIV Neg IgGs (median: 6.9, range: 5.1–7.9%, P = 0.0016) and non-ADCC IgGs (median: 7.18, range: 3.49–10.3% P < 0.0001) (**Figure 1D**).

The PMA stimulated ACH-2 also showed significant increase in the HIV gag DNA copies (median: 21,155 range: 18,300–22,103 copies/million cells) as compared to the unstimulated cells (median: 8,180 range: 7,996–8,210 copies/million cells) which were significantly decreased when ADCC IgGs (median: 3,445, range: 1,462–12,793 copies/million) were added. Whereas the number of DNA copies in non-ADCC IgGs (median: 17,452, range: 6,610–25,388 copies/million) were comparable with the copies observed in PMA stimulated ACH-2 + effector cells (P = 0.0023) (**Figure 1E**).

### Anti-HIV-1 ADCC Antibodies Lyse the ENV Stimulated HIV-Infected Autologous CD4+ T Cells

Further, we investigated whether the Env C-stimulated primary CD4+ T cell model also shows similar results. When the PBMCs (source of target and autologous effector cells) from the ADCC responders and non-responder study participants were stimulated with HIV Env C peptides, the frequencies of p24+ CD4+ cells increased significantly (median: 3.86%, range: 0.25–7.86%) in comparison with the frequencies in unstimulated cells (median: 0.65%, range: 0.10–3.3%) (P < 0.0001).

**Figure 3:**
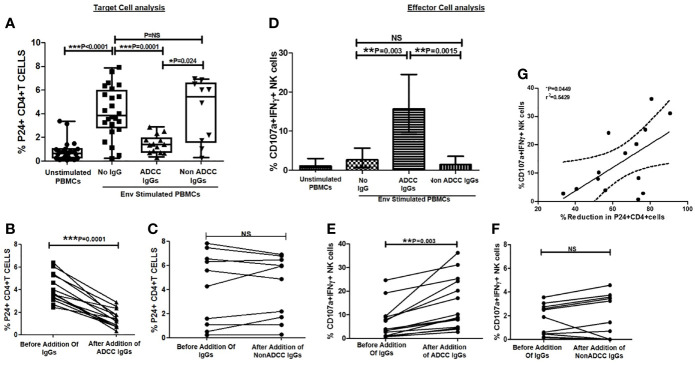
Ability of ADCC antibodies to lyse reactivated HIV-infected CD4+ T cells. **(A)** Scatter plot shows the frequencies of p24+CD4+ cells (on Y axis) before HIV ENV stimulation, after stimulation with HIV-1 C Env peptide pool and after addition of ADCC IgGs or non-ADCC IgGs(on X axis). The graph **(B)** shows frequencies of p24+CD4+ cells (on Y axis) before and after addition of ADCC IgGs (on X axis) in 14 paired samples (P = <0.0001). The graph **(C)** shows frequencies of p24+CD4+ cells (on Y axis) before and after addition of non-ADCC IgGs (on X axis) in 10 paired samples. (Wilcoxon Matched paired test) **(D)** The bar diagram shows CD107a and IFN*γ* secreting NK cells (on Y axis) before HIV ENV stimulation, after stimulation with HIV-1 C Env peptide pool, and after addition of ADCC IgGs and non-ADCC IgGs (on X axis). The graph **(E)** shows percent CD107a+IFN*γ*+ NK cells (on Y axis) before and after addition of ADCC IgGs (P = 0.003) and **(F)** non-ADCC IgGs in 14 and 10 paired samples respectively (Wilcoxon Matched paired test). **(G)** The scatter dot plot represents the positive association between the % reduction in p24+CD4+ cells (on X axis) with frequency of CD107a and IFN*γ* secreting NK cells (on Y axis) in ADCC responders. (Spearman correlation analysis, P < 0.05). NS, not significant.

After addition of ADCC IgGs to the Env-stimulated cells, these frequencies were decreased significantly (median: 1.4, range: 0.34–2.88, P = 0.0001) as compared to the frequencies observed in Env-stimulated cells (P = 0.0001) or when non-ADCC IgGs were added (median: 5.43, range: 0.28–6.92%) (P = 0.024) (**Figure 3A**). The paired analysis of the p24+CD4+ cell frequencies in Env C-stimulated cells before and after addition of ADCC IgGs or non-ADCC IgGs also showed significant reduction when the ADCC IgGs (N = 14) were added (P = 0.0001) (**Figure 3B**), whereas in case of addition of non-ADCC IgGs (N = 10) the frequencies did not change significantly (**Figure 3C**). The NK cell activation was also increased significantly after addition of ADCC IgG (median: 15.7, range: 5.2–36.2%) as compared with no antibody control (median: 2.66, range: 0.0–24.6%) (P = 0.003) and with non-ADCC IgG (median: 1.22, range: 0.153–12.6%) (P = 0.0015) (**Figure 3D**). The paired observations for_ NK cell activation before and after addition of ADCC IgGs showed significant increase after the addition (p = 0.003, **Figure 3E**), whereas this difference was insignificant in case of non-ADCC IgGs (**Figure 3F**). The increased frequencies of CD107A+IFN*γ*+NK cells in ADCC IgGs were positively associated with the percent reduction in frequency of p24+CD4+ cells (r^2^ = 0.54; P = 0.0449) (**Figure 3G**).

These experiments showed that the Env-C peptides could activate the primary CD4+ T cells, and the activated autologous NK cells could mediate killing of reactivated autologous CD4+ cells through ADCC.

### Reduction of Reactivated Resting Memory CD4+ Cells by ADCC

**Figure 4:**
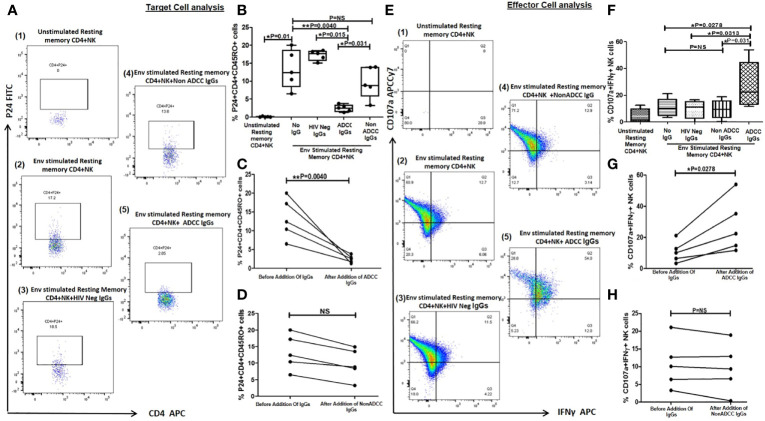
Lysis of reactivated resting memory CD4+ cells from HIV infected patients by ADCC. **(A)** The Representative FACS dot plots shows % P24+CD45RO+CD4+ cells (resting memory CD4) (P24 on Y axis and CD4 on X axis) in (1) unstimulated resting memory CD4 cells with effector cells (NK cells) (2), after HIV ENV C stimulation (3), after addition of HIV Neg IgGs (4), after addition of NonADCC IgGs, and (5) after addition of ADCC IgGs. **(B)** The bar diagram shows the frequency of p24+CD45RO+CD4+ cells (on Y axis) before HIV ENV C stimulation, after HIV ENV C stimulation, after addition of HIV neg IgGs, ADCC IgGs and non-ADCCC IgGs (on X axis). The paired line graph shows % p24+CD4+CD45RO+ cells (on Y axis) before and after addition of **(C)** ADCC IgGs (P = 0.004), and **(D)** before and after addition of non-ADCC IgGs. The data was analyzed with Wilcoxon matched paired T test. **(E)**. The representative dot plots shows CD107a (on Y axis) and INF*γ* (on X axis) secreting cells in (1) unstimulated resting memory CD4 cells with effector cells (NK cells) (2), after stimulation with Env C (3), after addition of HIV neg IgGs (4), after addition of non ADCC IgGs, and (5) after addition of ADCC IgGs. **(F)** The bar diagram shows percent CD107a and IFN*γ* secreting NK cells (on Y axis) before HIV ENV C stimulation, after ENV stimulation, after addition HIV neg IgGs, non-ADCC IgGs, and ADCC IgGs (on X axis). The paired graphs **(G)** shows frequencies of CD107a+IFN*γ*+ NK cells (on Y axis) before and after addition of ADCC Abs (P = 0.0278) (Wilcoxon matched paired T test) and **(H)** before and after addition of non-ADCC IgGs. NS, not significant.

Further we wanted to assess whether the latent reservoir of HIV, resting memory CD4+ T cells can be activated by Env-C antigen and whether these reactivated cells are lysed by the ADCC mediating anti-HIV antibodies. For this we used the earlier described flow based assay to determine reduction in Env-C stimulated resting memory cells after addition of ADCC IgGs. The frequencies of reactivated P24+CD45RO+CD4+ cells were significantly increased after stimulation with HIV-1 C Env peptides (median: 10.4, range: 6.48–20%) as compared to unstimulated control (median: 0.046, range:0–0.24%) (**Figure 4B**). The frequencies of these cells were significantly reduced after addition of ADCC IgGs (median: 2.52, range: 1.2–3.79%) (P = 0.004), whereas the frequencies after the addition of non -ADCC IgGs (median: 8.84, range: 3.2–14.9%) (P = 0.031) or HIV neg IgGs (median: 17.95, range: 15.1–18.5%) (P=0.015) were comparable to the Env-C reactivated cells (**Figure 4B**). The paired analysis also showed significant reduction in frequencies of P24+CD45RO+CD4+ cells in case of ADCC IgGs as compared to the non-ADCC IgGs (**Figures 4C, D** **)**.

Significantly higher NK cell activation was observed when ADCC IgG was added (median: 22.4, range: 11.7–54%) (P = 0.027) as compared to the reactivated cells without any antibody (median: 10.1, range: 3.32–21.1%), in case of addition of HIV Neg IgG (median: 11.3, range: 0.07–16.6%) (P = 0.0313) or non-ADCC IgG (median: 9.37, range: 0.38–18.9%) (P = 0.031) (**Figure 4F**). The NK cell activation before and after addition of ADCC IgGs (**Figure 4G**) and non-ADCC IgGs (**Figure 4H**) showed significant increase only when ADCC IgGs were added.

## Discussion

The persistence of HIV in the infected individual in spite of effective ART is a major hurdle in the cure of HIV. The shock and kill approach to reactivate the latent reservoir and then killing of the same by immune mechanisms are being tried to reduce the reservoir size and obtain functional cure in HIV infection. The effective immune mechanism might require immune boosting for prompt lysis of the reactivated reservoirs (16). The HIV-1 broadly neutralizing antibodies (bNAbs) have shown a possible role in reduction of latent reservoir by Fc-mediated mechanism such as ADCC (17–21). However, development of escape mutants can cause resistance against bNAb monotherapy. Hence the polyclonal ADCC mediating antibodies could be possibly useful in broad recognition of infected cells and their lysis

In the present study, we examined role of anti-HIV-1 polyclonal antibodies mediating ADCC in NK cell mediated lysis of the reactivated reservoir. We used purified IgGs from the plasma of ADCC responders and non-responders as a source of polyclonal antibodies and flow based assay to estimate the frequencies of p24 expressing target cells as a measure of reactivation and lysis after addition of antibodies and NK cells. Particularly, in comparison to other culture-based assays such as Quantitative Virus Outgrowth Assay (QVOA) and inducible transcription assays that follow a long protocol, this flow-based assay is simple and has a shorter stimulation period and requires less number of cells (22).

We have used flow based assay described earlier by Lee et al. (15) and modified further to accommodate the use of *ex vivo* unaltered primary CD4 cells and sorted resting memory CD4 cells to assess the lysis of reactivated latent reservoir, which will be closer to the *in vivo* situation. In our study the comparison between the purified IgG from ADCC responders and non-responders showed conclusively that only ADCC mediating antibodies can reduce the Env C-reactivated p24 expressing cells in three different cell types, the latent reservoir cell line; ACH 2 (**Figure 1D**), the reactivated primary HIV infected CD4+ T cells (**Figures 3B, C** **)** and resting memory CD4+ T cells (**Figures 4C, D** **)** from HIV infected individuals. These observations indicate that the lysis of the reactivated cells is a property of ADCC mediating anti-HIV antibodies only. The significant increase in NK cell activation in parallel to the reduction in p24 expressing target cells indicates that the lysis of the Env-reactivated HIV-1-infected cells was mediated by ADCC and NK cells (**Figures 1D**; **3E**, **4G**). The insignificant or lack of NK cell activation when the antibodies not mediating ADCC were added further supports the role of ADCC mediating antibodies in reduction in reactivated latent reservoir.

We used intracellular p24 expression as a marker for reactivation of target cells and a parameter for evaluating ADCC-mediated lysis, but due to the small number of cells available for the experiment, we were unable to determine the expression of ENV on reactivated target cells. This is one of the study’s limitations. However, Lee et al. showed that intracellular p24 expressing ACH-2 cells have expressed ENV antigen on their surface (15), suggesting that p24 expressing target cells in our study should also express ENV antigen on the cell surface.

It is known that the full HIV-1 replication in primary T cells takes at least 24 h (23); hence the reduction in the p24 expressing cells in a short stimulation cycle used in this study could be ADCC mediated rather than suppression of new infection by antibody neutralization. The broadly neutralizing antibodies could lyse reactivated latent cells through the FC mediated involvement of NK cells (24–26). We could not test the HIV neutralization ability of the ADCC antibodies used in this study which is one of the limitations. However, if so, the combining activity of these antibodies can be useful in the blockade of viral infectivity by neutralization and lysis of infected cells through ADCC mediated recruitment of innate effector cells such as NK cells.

It is also known that the antigen bound antibodies with the immune cells can act as a signal to improve the immune responses at local and systemic levels (27). Such cell surface bound immune complex recognition by plasmacytoid and myeloid dendritic cells has shown to improve the antigen uptake and presentation, allowing induction of stronger humoral and cellular antiviral immune responses (28). Hence the ADCC mediating antibody could possibly work as immune booster also in addition to their primary function of activation of NK cells by binding with surface expressed antigen.

The primary resting memory CD4 cells from different individuals can indicate the heterogeneity of HIV reservoirs. We also showed range of frequencies of P24+CD4+ cells from 0 to 0.24% in resting memory cells indicating the variability in HIV reservoirs in different individuals similar to previous reports (29) indicating the importance of use of primary cell model.

**Figure 5:**
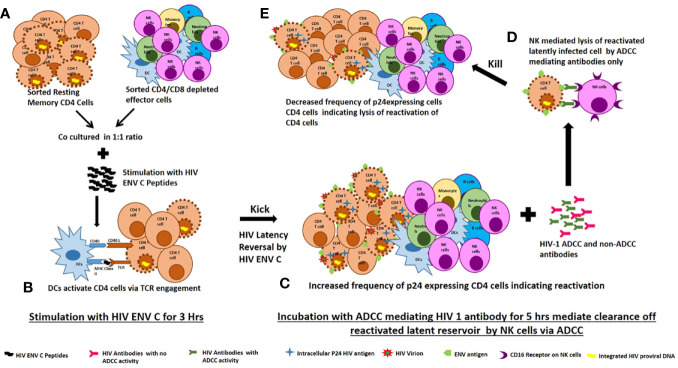
Proposed mechanism of ENV-C reactivation of latent HIV reservoirs and their killing by ADCC mediating lysis by NK cells. **(A)** Sorted resting memory cells and sorted CD4/CD8 depleted effector cells were cultured at 1:1 ratio and stimulated with HIV ENV C peptides. **(B)** Dendritic cells specifically present MHC II bound antigenic peptide to HIV specific CD4 T cells *via* T cell receptor engagement. Activation of the T-cell receptor (TCR) induces multiple signal transduction pathways leading to the ordered nuclear migration of the HIV transcription initiation factors NF-*κ*B and P-TEFb activate latent HIV proviruses through an ERK-dependent pathway. **(C)** HIV ENV C stimulation increased the frequency of p24 expressing CD4 cells indicating reversal of latency. **(D)** Addition of ADCC mediating antibodies mediate clearance reactivated latently infected cells by NK cells through ADCC. **(E)** The reduction in frequency of p24 expressing CD4 cells after addition of ADCC mediating antibodies indicates lysis of reactivated latently infected cells.

A number of studies have used different LRAs for reactivation. Many recent studies have shown that LRAs have a detrimental effect on the activity of CD8+ T cells and NK cells, resulting in impaired clearance of HIV infected cells (30, 31). Since HIV-1 preferentially infects activated CD4 T cells (32) and activation of latently infected memory CD4 T cells by their cognate antigens is more specific, we propose that HIV antigens can be used as latency reversing agent (33). To the best of our knowledge, this approach for use the Env C peptides as latency reversing agent in resting memory cells is proposed only in our previous study (12) and the present study. We propose that, dendritic cells specifically present MHC II bound antigenic peptide to HIV specific CD4 T cells *via* T cell receptor engagement. Activation of the T-cell receptor (TCR) induces multiple signal transduction pathways leading to the ordered nuclear migration of the HIV transcription initiation factors NF-*κ*B and P-TEFb as with maximal accumulation as early as 30 min after stimulation and hence able to activate latent HIV proviruses through an ERK-dependent pathway in this short period of stimulation (34). This hypothesis has been explained in **Figure 5**.

We showed that both primary and resting memory CD4+ T cells from ART naïve HIV infected individuals could be reactivated with envelope C antigen. We also confirmed that the resting memory CD4+ T cells had very low p24 expression as compared to the total CD4+ T cells before activation. However, further experiments are required to assess the ability of HIV ENV C to reactivate the primary CD4 cells and resting memory CD4 cells from ART-treated HIV infected individuals with suppressed viremia. The results of RV 144 HIV vaccine trial also suggest that the combination of Env immunogens induced antibodies with ADCC activity that correlated with protection in individuals showing low IgA response (35, 36). It would be interesting to assess the ability of Env to boost the ADCC response.

We used short stimulation flow based assay using primary memory CD4 cells to determine ADCC mediated killing of the reactivated latent reservoir. In the absence of an *in vivo* model, this assay has a potential in rapid assessment of LRAs for reservoir reduction and hence can be further explored. Similarly this assay will also be useful in the assessment of the newly designed antibodies for their ability to kill reactivated HIV reservoirs.

The major limitation of ADCC mediated lysis of latent reservoir is the fact that these antibodies are reduced in suppressive ART and hence may not be effective in recruiting NK cells for lysis of reactivated infected cells. In such case, the ADCC mediating antibodies can be passively transferred as an immunotherapy which might overcome the absence of antibody in ART treated individual. The observations of our study will be useful in further exploration of the role of ADCC mediating antibodies in functional cure in HIV infection. Future research on the epitopes that are recognized by ADCC mediating anti-HIV antibodies could expand our understanding and demonstrate future promise in the design of antibody-mediated immunotherapy for latent HIV reservoir eradication.

## Data Availability Statement

The original contributions presented in the study are included in the article/**Supplementary Material**. Further inquiries can be directed to the corresponding author.

## Ethics Statement

The studies involving human participants were reviewed and approved by Institutional Ethics Review Board of ICMR-National AIDS Research Institute. The patients/participants provided their written informed consent to participate in this study.

## Author Contributions

PS contributed to the design of the study, methodology, and analysis of data, and writing the manuscript. RB contributed to the enrollment of study participants in the study. AS contributed to study design, data analysis, and review of the manuscript. MT conceptualized the study, data curation, review, and finalization of the manuscript. All authors contributed to the article and approved the submitted version.

## Funding 

This research was supported by intramural funding from ICMR–National AIDS Research Institute, Pune.

## Conflict of Interest

The authors declare that the research was conducted in the absence of any commercial or financial relationships that could be construed as a potential conflict of interest.

## Supplementary Material

Supplementary Figure 1 Gating Strategy for flow based assay using ACH2 cell line **(1)**. The lymphocytes were gated based on FSC/SSC scatter **(2)**. The NK cells were then identified as CD3−CD56+ cells **(3)**.The ACH2 cells were identified as CD3− cells as they do not express CD4 and CD8 markers and **(4)** assessed for intracellular P24 expression.

Supplementary Figure 2 Gating Strategy for flow based assay using HIV ENV C-activated HIV Infected CD4+ T cells. **(A)** The FACS dot plot shows the gating strategy for CD4+ cells and NK cell **(1)**. The lymphocytes were gated on the basis of forward and side scatter **(2)**. CD3−CD56 dim+ cells gated as NK cells and further assessed for secretion of CD107a and IFN*γ* secretion **(3)**. CD3+CD8− cells were gated as CD4+ cells and drilled down further to determine the intracellular p24 expression using fluorescence minus one (FMO) for p24 as control **(4)**.

Supplementary Figure 3 Gating Strategy for flow based assay using Resting Memory CD4+ Cells **(1)**. The lymphocytes were gated on the basis of forward and side scatter. Next **(2)** CD4−CD8− cells were gated effector NK cells and collected in one tube, and CD4+CD8− cells were gated as CD4 cells. These CD4+ cells were drilled down for sorting CD45RO+CD4+ cells using **(3)** fluorescence minus one (FMO) for CD45RO as control and **(4)** collected in the second tube.
