# Supplementary figures and images for: Anti-HIV-1 ADCC and HIV-1 Env Can Be Partners in Reducing Latent HIV Reservoir
Source: Front Immunol. 2021 Apr 30;12:663919. doi: 10.3389/fimmu.2021.663919 (PMC8119992; doi:10.3389/fimmu.2021.663919)

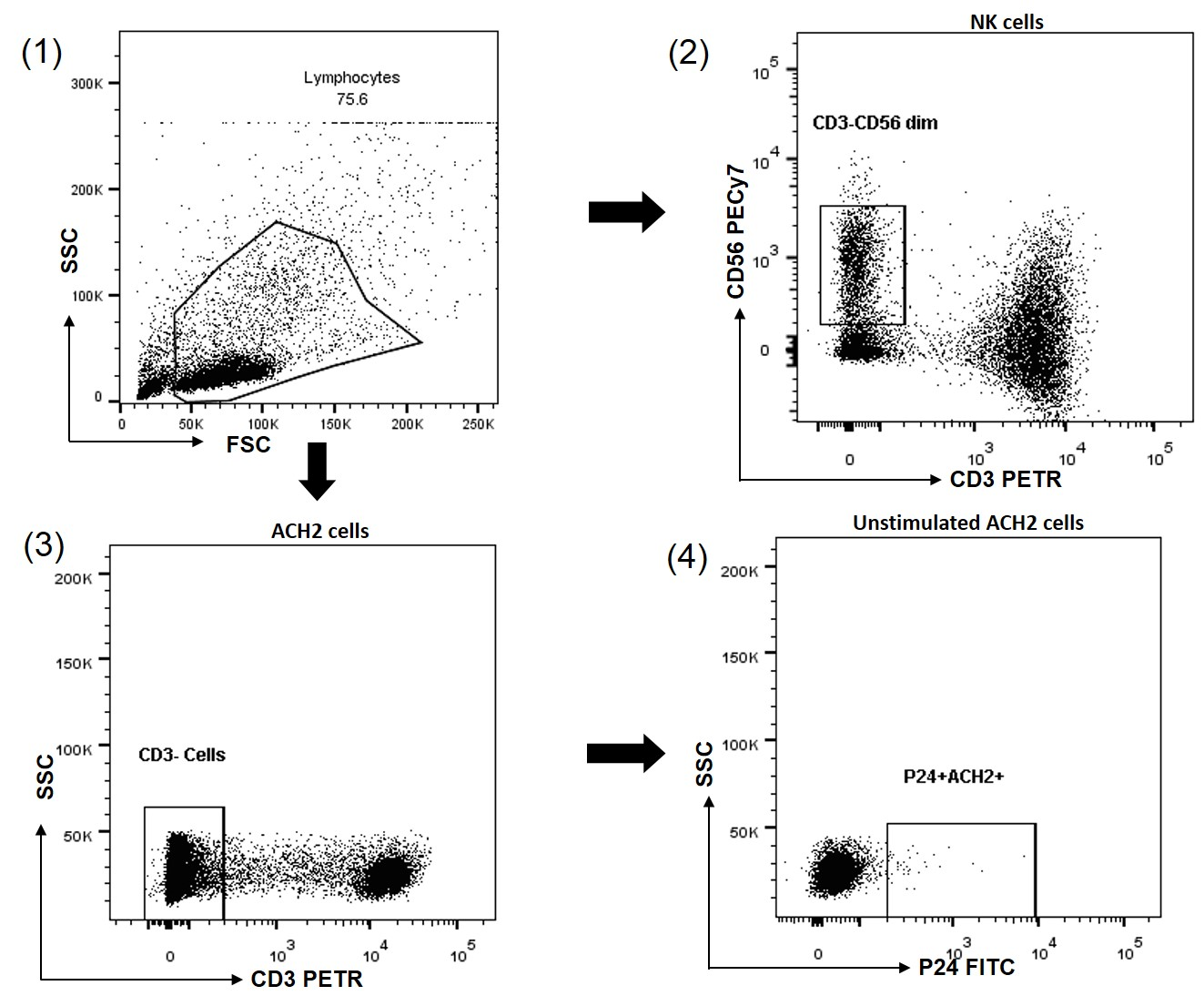

Supplement: Supplementary Figure 1 — Gating Strategy for flow based assay using ACH2 cell line (1). The lymphocytes were gated based on FSC/SSC scatter (2). The NK cells were then identified as CD3−CD56+ cells (3).The ACH2 cells were identified as CD3− cells as they do not express CD4 and CD8 markers and (4) assessed for intracellular P24 expression. [file Image_1.tif]

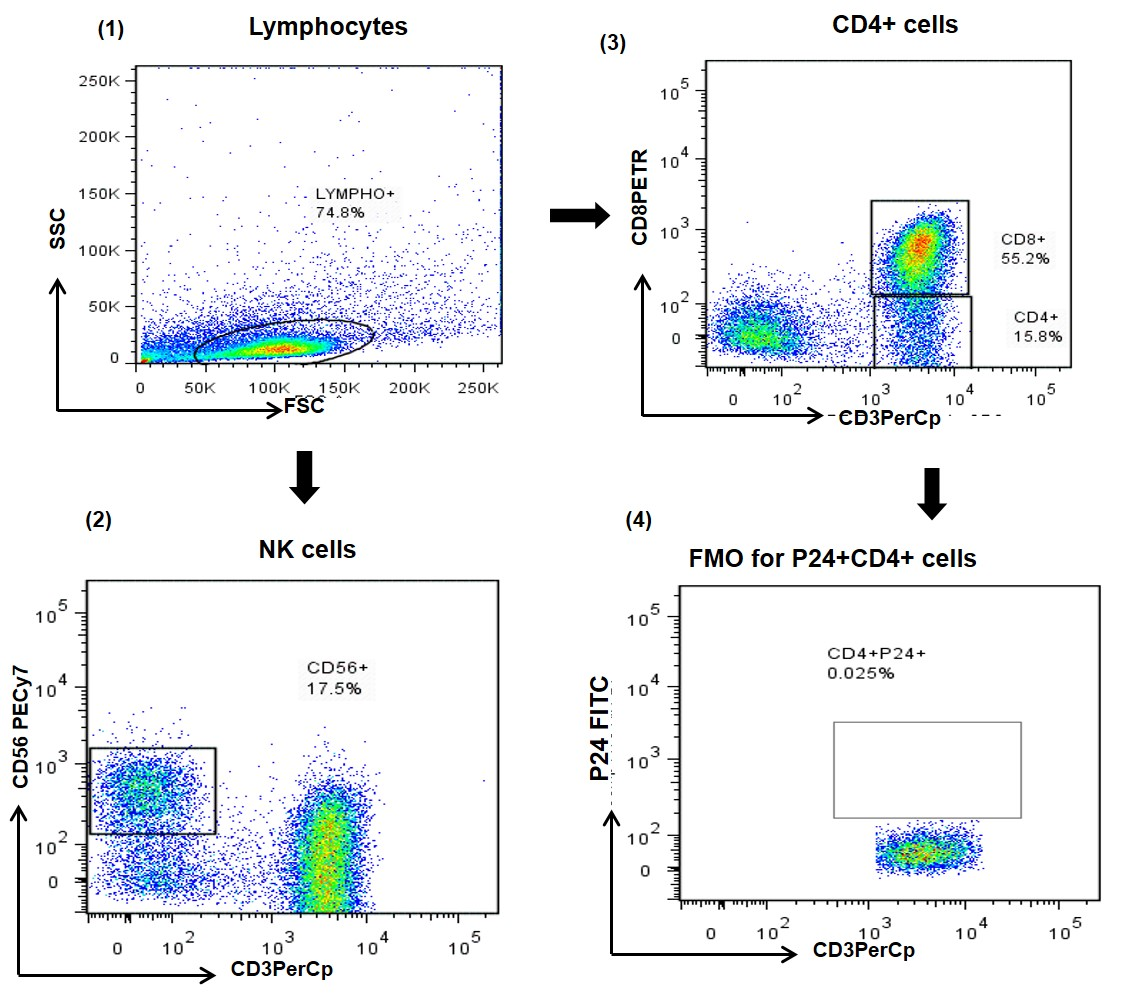

Supplement: Supplementary Figure 2 — Gating Strategy for flow based assay using HIV ENV C-activated HIV Infected CD4+ T cells. (A) The FACS dot plot shows the gating strategy for CD4+ cells and NK cell (1). The lymphocytes were gated on the basis of forward and side scatter (2). CD3−CD56 dim+ cells gated as NK cells and further assessed for secretion of CD107a and IFNγ secretion (3). CD3+CD8− cells were gated as CD4+ cells and drilled down further to determine the intracellular p24 expression using fluorescence minus one (FMO) for p24 as control (4). [file Image_2.tif]

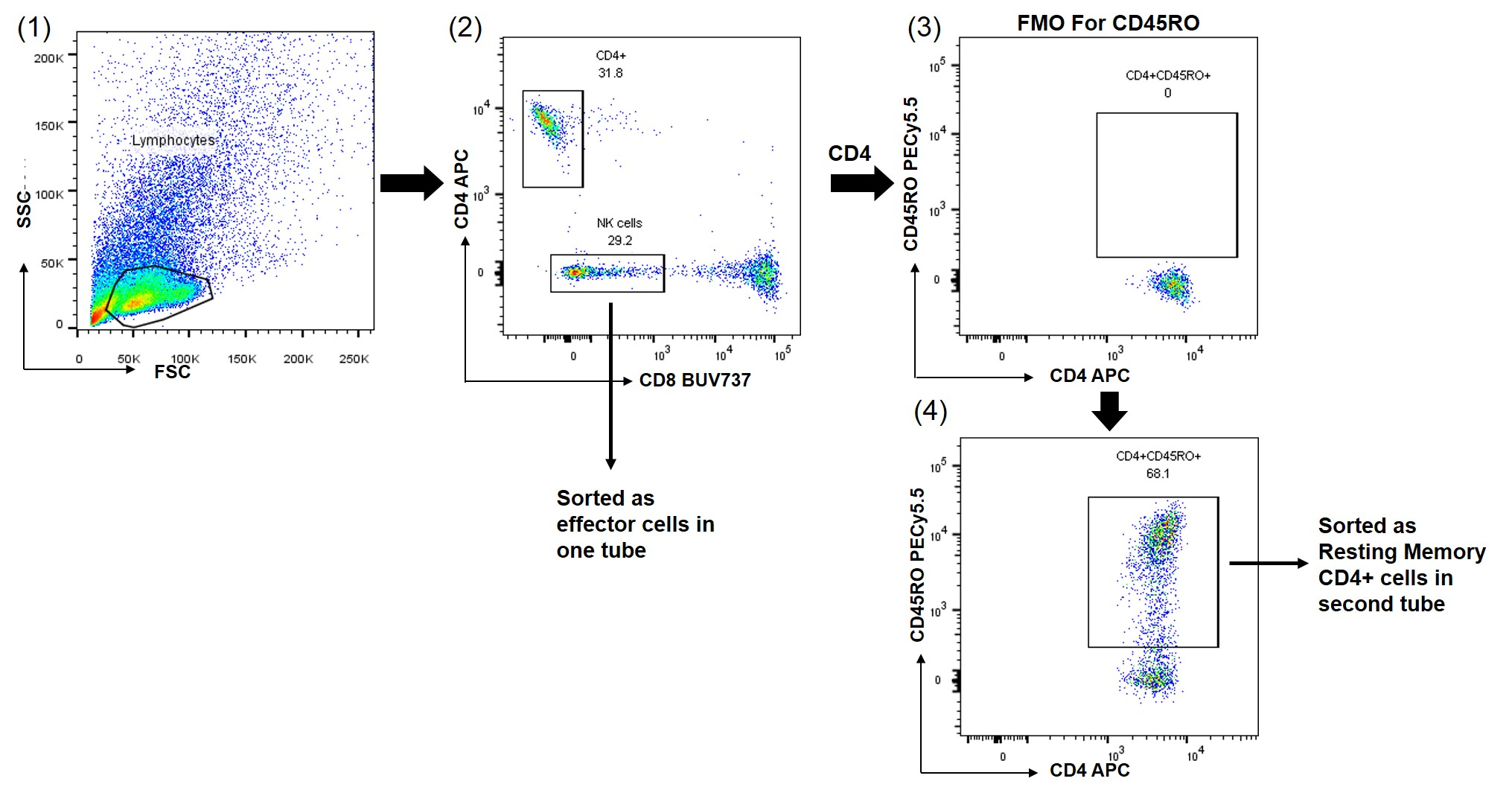

Supplement: Supplementary Figure 3 — Gating Strategy for flow based assay using Resting Memory CD4+ Cells (1). The lymphocytes were gated on the basis of forward and side scatter. Next (2) CD4−CD8− cells were gated effector NK cells and collected in one tube, and CD4+CD8− cells were gated as CD4 cells. These CD4+ cells were drilled down for sorting CD45RO+CD4+ cells using (3) fluorescence minus one (FMO) for CD45RO as control and (4) collected in the second tube. [file Image_3.tif]
